# Long-Term Follow-up of HPV Infection Using Urine and Cervical Quantitative HPV DNA Testing

**DOI:** 10.3390/ijms17050750

**Published:** 2016-05-17

**Authors:** Alex Vorsters, Severien Van Keer, Samantha Biesmans, Annick Hens, Ilse De Coster, Herman Goossens, Margareta Ieven, Pierre Van Damme

**Affiliations:** 1 Centre for the Evaluation of Vaccination, Vaccine & Infectious Disease Institute, University of Antwerp, 2610 Wilrijk, Belgium; severien.vankeer@uantwerpen.be (S.V.K.); samantha.biesmans@uantwerpen.be (S.B.); annick.hens@uantwerpen.be (A.H.); ilse.decoster@uantwerpen.be (I.D.C.); pierre.vandamme@uantwerpen.be (P.V.D.); 2 Laboratory of Medical Microbiology, Vaccine & Infectious Disease Institute, Faculty of Medicine and Health Sciences, University of Antwerp, 2610 Wilrijk, Belgium; herman.goossens@uantwerpen.be (H.G.); greet.ieven@uza.be (M.I.); 3 Clinical Microbiology, Antwerp University Hospital, 2650 Edegem, Belgium

**Keywords:** urine, HPV DNA, vaccination, HPV testing, self-sampling

## Abstract

The link between infection with high-risk human papillomavirus (hrHPV) and cervical cancer has been clearly demonstrated. Virological end-points showing the absence of persistent HPV infection are now accepted as a way of monitoring the impact of prophylactic vaccination programs and therapeutic vaccine trials. This study investigated the use of urine samples, which can be collected by self-sampling at home, instead of cervical samples for follow-up of an HPV intervention trial. Eighteen initially HPV DNA-positive women participating in an HPV therapeutic vaccine trial were monitored during a three-year follow-up period. A total of 172 urine samples and 85 cervical samples were collected. We obtained a paired urine sample for each of the 85 cervical samples by recovering urine samples from six monthly gynaecological examinations. We performed a small pilot study in which the participating women used a urine collection device at home and returned their urine sample to the laboratory by mail. All samples were analyzed using quantitative real-time HPV DNA PCR. A good association (κ value of 0.65) was found between the presence of HPV DNA in urine and a subsequent cervical sample. Comparisons of the number of HPV DNA copies in urine and paired cervical samples revealed a significant Spearman rho of 0.676. This correlation was superior in women with severe lesions. The HPV DNA results of the small pilot study based on self-collected urine samples at home are consistent with previous and subsequent urine and/or cervical results. We demonstrated that urine sampling may be a valid alternative to cervical samples for the follow-up of HPV intervention trials or programs. The potential clinical value of urine viral load monitoring should be further investigated.

## 1. Introduction

A link between infection with high-risk human papillomavirus (hrHPV) types and cervical cancer was suggested in 1977, and the presence of hrHPV as the necessary cause of invasive cervical cancer was confirmed in 1999 [1,2]. Bi- and quadrivalent vaccines were introduced in the past 10 years. A nonavalent vaccine was developed recently, and it is registered in the US and EU. These vaccines effectively prevent persistent infection and precancerous lesions [3,4,5,6].

The potential role of viral end-points in facilitating the follow-up of HPV prophylactic vaccine trials has received much attention in the last several years. The International Agency for Research on Cancer (IARC) and the World Health Organization (WHO) reported that persistent infection of six months or longer likely acts with high fidelity as a surrogate for advanced disease/cancer [7]. In addition, hrHPV viral infection is essential in nearly 100% of cervical cancer cases. Therefore, the prevention of HPV infection should also prevent the development of premalignant and malignant disease. Vaccine efficacy using the viral end-point was similar to vaccine efficacy obtained by the Cervical Intraepithelial Neoplasia Grade two or worse (CIN2+) end-point for HPV vaccine types. A similar or lower temporary vaccine efficacy was observed for non-vaccine types because of partial cross-protection [8,9,10,11,12,13]. The IARC working group reported three major benefits of the use of type-persistent infection instead of CIN2+ as the end-point in trials. First, it permitted a 10-fold reduction in sample size. Second, the follow-up phase after the final dose could be shortened by 2–3 years. Finally, the complexity and management of the study could be substantially reduced [7].

Viral end-points for HPV therapeutic vaccine trials have been less extensively discussed and studied in long-term follow-up settings. However, the efficacy of an HPV therapeutic vaccine may be demonstrated using the clearance rate of a vaccinated cohort compared with the clearance rate of an unvaccinated control cohort. Clearance is defined as the sustained (6–12 months) absence of viral infection, which is consistent with the use of the viral end-point in prophylactic vaccine trials.

The use of viral end-points provides another major advantage. The field of cervical cancer screening demonstrated that HPV DNA PCR testing of self-collected *versus* clinically collected samples exhibited a similar sensitivity [14], which offers opportunities to further reduce the complexity of these studies and increases the likelihood of participation in follow-up programs.

This study demonstrated the performance of HPV DNA urine testing (including viral load) during a three-year follow-up of a cohort of 18 HPV16- and/or HPV18-infected women and compared the quantitative HPV DNA urine results to quantitative HPV DNA results obtained using paired cervical samples. This study investigated whether viral end-point information derived from self-collected urine samples corresponded to the viral end-point information derived from cervical samples. In a small pilot study, we also investigated the use of a first-void urine collection device (Colli-Pee^TM^, Novosanis, Wijnegem, Belgium) for home sampling.

## 2. Results

### 2.1. HPV DNA Results from Urine and Cervical Samples

Table 1 provides a summary of the qPCR results. A total of 172 urine samples and 85 cervical samples were collected and tested in this cohort. The volumes of the collected first-void urine samples were very diverse. The collected urine volume was 7–86 mL depending on the participant and visit (Figure S1). The urine volume of Colli-Pee^TM^-collected first-void urine samples was 8 to 18 mL. We detected more copies of human DNA in urine samples than cervical samples (Table 1). We detect significantly fewer copies of HPV DNA in urine compared with cervical samples (*p* < 0.001). We also detected fewer HPV18 DNA copies in urine and cervical samples compared with HPV16 DNA.

Table 2 and Table 3 show a two-by-two table of the agreement of HPV16 and HPV18, respectively, at month (M)0, 6, 12, 18, and 24. The κ value for HPV16 was higher than HPV18 (twice the statistical significance). Fewer false-negative HPV16 results were observed compared with false-negative HPV18 results when HPV DNA was used in the cervical sample as a reference.

**Figure 1:**
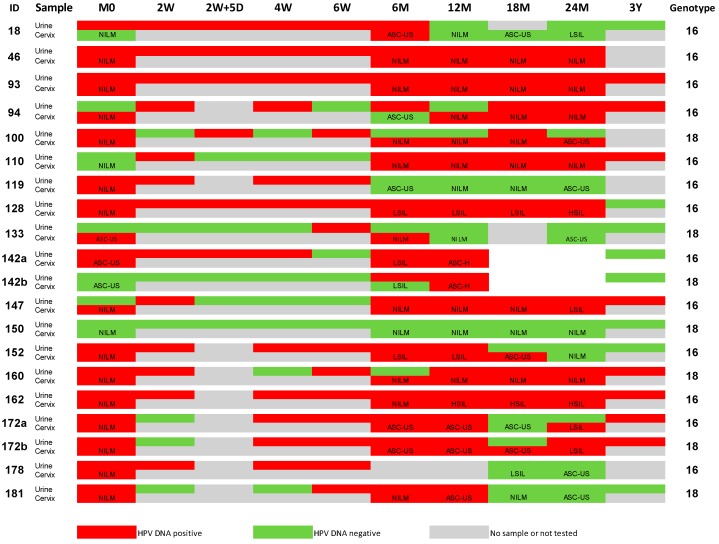
Schematic overview of HPV DNA results over the three-year follow-up period. The results obtained for home-collected samples are not included, but these results are provided in Figure S4. The sample ID of the participants is shown in the left column, and the HPV DNA results are provided for each participant; red: positive, green: negative. The top row also indicates the time-point of sampling during follow-up. M0 (month 0, start of the trial), W: week, D: days, Y: years. The right column indicates the HPV genotype—16 or 18. If a participant had HPV16 and HPV18 co-infection, the results for both genotypes are provided (e.g., 142a and 142b). The cytological results are provided for each collected cervical sample: NILM (Negative for Intraepithelial Lesion or Malignancy), ASC-US (Atypical Squamous Cells of Unknown Significance), ASC-H (Atypical Squamous Cells of Undermined Significance cannot exclude High-Grade Intraepithelial Lesions), LSIL (Low-grade Squamous Intraepithelial Lesions) or HSIL (High-grade Squamous Intraepithelial Lesions).

Figure 1 shows a complete overview of HPV DNA status in a cohort of 18 women over the three-year follow-up. Participant 172 exhibited HPV16 and HPV18 co-infection at the start of the study. Participant 142 became positive for HPV18 at M18. All available urine samples were tested for HVP16 and HPV18 for both of these women. These women explain the 20 HPV DNA results at the start of the study (at M0).

### 2.2. Correlation of HPV DNA Levels in Urine and Cervical Samples

**Figure 2:**
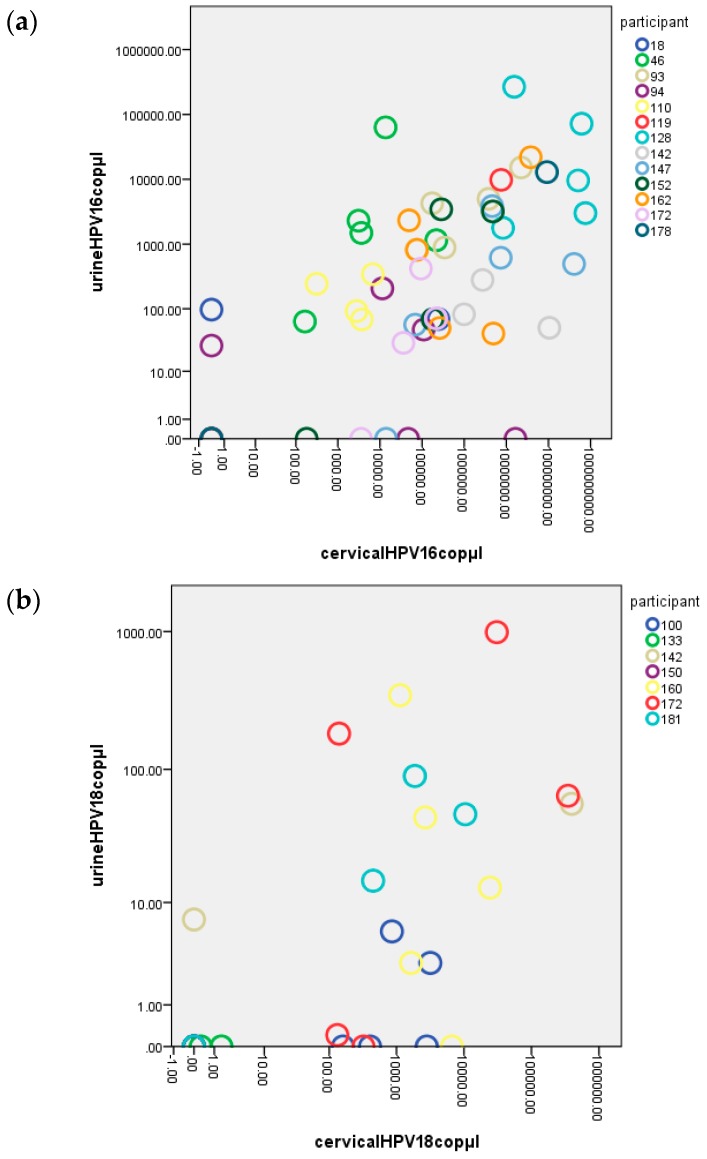
HPV copies in urine samples *versus* cervical samples: HPV16 (**a**) and HPV18 (**b**).

The obtained viral copy numbers for each of the participants at all data points are provided as supplementary information in Figure S2a–t. Figure 2 shows a dot plot of all quantitative data obtained for HPV16 (Figure 2a) and HPV18 (Figure 2b) at M0, 6, 12, 18, and 24 using urine and paired cervical samples. The data were not normally distributed (not shown), but we did find a significant (*p* < 0.001) Spearman’s rho correlation between HPV copies detected in urine and subsequent cervical samples of 0.676 and 0.695 for HPV16 and HPV18, respectively.

### 2.3. Impact of Cytology on the Correlation of Urine versus Cervical

The cytological results were also available for each cervical sample (Figure 1). NILM (Negative for Intraepithelial Lesion or Malignancy) or ASC-US (Atypical Squamous Cells of Undetermined Significance) were detected in most samples. However, eight participants had one or more cervical samples that exhibited LSIL (Low-grade Squamous Intraepithelial Lesions) or HSIL (High-grade Squamous Intraepithelial Lesions) (e.g., 18, 128, 142, 147, 152, 162, 172, or 178). LSIL was detected in the cervical sample of participants 18 (at M24) and 178 (at M18), but no HPV16 was detected in cervical or urine samples. However, hrHPV DNA rather than HPV16 and HPV18 was present in the cervical samples from both cases. In the present study, urine samples were not tested for hrHPV DNA other than the HPV16 and HPV18 types. Urine samples were also HPV16- or HPV18-positive for all other LSIL or HSIL cervical samples. A high number of viral HPV copies of ID128 was detected in cervical and urine samples (Figure 2Sh). Examination of the concordance for LSIL and HSIL samples specific for HPV16 DNA revealed a stronger correlation between urine HPV16 copies/µL DNA extracts and cervical HPV DNA copies/per cell than between urine HPV16 copies/µL DNA extract and cervical HPV DNA copies/µL of DNA extract (Spearman rho of 0.892 and 0.842, respectively, *p* < 0.001). A Pearson correlation of *R* = 0.793 (*p* = 0.004) was achieved after a logarithm to base 10 transformation of urine HPV16 copies/µL and cervical HPV16 copies/cell (see also Figure S3).

### 2.4. Results for Home-Collected Urine Samples

Ten women also agreed to participate in a small pilot study in which they received a first-void urine collection device (Colli-Pee^TM^) for use at home. Figure S4 shows that the results of the Colli-Pee^TM^ were concordant with previous and subsequent urine and cervical samples collected at the clinic. The HPV copy number in the Colli-Pee^TM^-collected urine samples were also in the range of the copy numbers found in previous and subsequent urine samples (Figure S2a,b,d,e,f,h,j,l,o,p).

## 3. Discussion

This study is, to our knowledge, the first longitudinal follow-up study utilizing type-specific quantitative PCR to compare HPV DNA in cervical and urine samples in HPV16- and/or HPV18-positive women.

### 3.1. Human DNA Detection in Urine

Notably, all 172 urine samples contained detectable human DNA, compared with previous studies of HPV DNA in urine [15]. Human DNA is not an ideal confirmation of good sample storage or sample processing because HPV DNA may decay faster than human DNA [16]. However, human DNA-negative urine samples may indicate suboptimal sample collection, handling or extraction methods and result in a lower HPV prevalence [15].

Our results were compared with the PapU studies from Payan *et al.* and Ducanelle *et al.* [17,18]. These investigators also examined paired urine and cervical samples in a cohort of women consulting a gynaecologist at three university hospitals. The impact of the DNA extraction method on HPV DNA recovery and lack of preservative has been reported previously [16]. We demonstrated that the 1 mL centrifugation step without preservative that is used by the French authors yielded more copies of human DNA than the other methods. However, an additional amount of DNA, including so-called cell-free DNA, was recovered when a DNA preservative was used. If we convert our results, which were expressed as DNA copies per µL DNA extract, to DNA copies per mL of urine, we find that the use of our method yielded 470 to 1.8 × 10^6^ copies of human DNA per mL of urine, providing a higher concentration than the reported 0 to 3 × 10^5^ cells reported in the French PapU study. We also found more copies of human DNA in urine samples than cervical samples, which may be due to the enhanced processing of the samples. Urine samples were transferred to a BD vial (BD ProbeTecTM Urine Preservative Transport Kit, BD Benelux, Erembodegem, Belgium) within 30 min and concentrated via Amicon filtration prior to Nuclisense Easy Mag extraction. This process, in combination with the presence of a nuclease inhibitor in the BD vial, ensures the recovery of all DNA present, including cell-free DNA. The amount of human DNA in cervical samples is linked to the number of cells collected by a cytobrush and the subsequent recovery for liquid-based cytology (LBC). All non-cell associated DNA is removed in the process of LBC preparation, which contributes to the detection of less human DNA in cervical samples.

### 3.2. Detection of HPV DNA in Urine

We used a relatively sensitive urine DNA recovery/detection method, as demonstrated by the recovery of human DNA in all urine samples. However, numerous enhancements may increase the analytical sensitivity of HPV DNA detection in urine. Table 2 and Table 3 show that the κ agreement for HPV16 and HPV18 positivity in urine and paired cervical samples was, respectively, good (κ = 0.683) and fair (κ = 0.510). The mean and average copies/µL DNA extract of HPV16 in cervical and urine samples was much higher than that of HPV18. Ducancelle *et al.* [18] reported, and this study confirmed, that the most discrepant results between cervical and urine samples are observed when low HPV copy numbers in urine and/or cervical samples are reported, which indicates that the amount of HPV DNA is near the limit of detection. The French study also reported that copy numbers were higher in women with HSIL [18]. This result is consistent with our findings. We also found more HPV DNA copies in paired urine and cervical samples provided by women with an LSIL or HSIL lesion, and the observed Spearman rho between urine HPV DNA copies/µL DNA extract and cervical HPV DNA copies/µL DNA extract was 0.842 for this subset of samples, indicating a very strong correlation.

Urine collection could still be improved by reducing the volume of the first-void [19], but we found concordant positive HPV16 or HPV18 DNA results in all urine and paired cervical samples from women with HSIL and LSIL lesions induced by HPV16 or HPV18. A discrepant result for HPV16 was observed for participant 172 at M24. However, a low copy number was also reported in the cervical sample, and we do not know which HPV genotype was responsible for the LSIL lesions. Notably, the cervical sample was also HPV16 negative at M18. This participant had a HPV16- and HPV18-positive urine sample at the three-year follow-up.

The required clinical sensitivity of hrHPV detection in urine must still be determined for cervical cancer screening purposes. However, the higher copy numbers found in HSIL and LSIL lesions suggests that the highest analytical sensitivity will not be required. Nonetheless, among the 25 HSIL-diagnosed women in the French PapU study, six apparently had HPV negative urine. However, there are indications that the collection, preservation and or processing of the samples did not result in a high analytical sensitivity in this study. Payan *et al.* reported that 15% of the urine samples lacked detectable human DNA and had an 8% lower prevalence of HPV DNA in urine compared with cervical samples [17]. This lower HPV DNA prevalence contradicts several recent studies that reported an increased number of urine samples that were positive for HPV DNA compared with cervical samples. We demonstrated that the use of our in-house DNA preservative and DNA extraction method yielded a greater amount of HPV DNA-positive urine samples compared with the paired cervical samples in a previous study of 540 young Colombian women [20]. A higher HPV DNA prevalence was also observed in the collected urine samples compared with the HPV DNA results obtained from paired cervical samples in women in the UK [21].

The need for further clarification of the application of a highly sensitive method for HPV urine testing was also addressed in the setting of HPV vaccination impact monitoring [22]. We may detect low levels of HPV DNA using a very analytical and sensitive method, which may not have clinical significance. However, if urine is used to establish a virological endpoint, then the assessment of the total absence of HPV DNA may justify the use of a highly sensitive detection method.

### 3.3. Determination of the HPV DNA Viral Load in Urine

The potential clinical or prognostic role of viral load assessment during the follow-up of an HPV infection remains a challenging topic [23,24]. A discussion of the value of viral load is beyond the scope of this study, but we discuss the observed correlation between HPV DNA levels in urine and paired cervical samples and issues that may be encountered with the use of HPV DNA levels in urine.

Very few studies have investigated viral load or HPV DNA levels in urine. To our knowledge, no other studies have elaborated on the results of Payan *et al.* who reported in 2007 that the levels of viral load in cervical samples were significantly correlated to the levels of HPV DNA in urine [17]. We also found a correlation between the amount of HPV DNA detected in a cervical sample and the amount of HPV DNA detected in a paired first-void urine sample. Our results provided a strong Spearman rho correlation of >0.67 between the HPV16 and HPV18 copies detected in urine and paired cervical samples (Figure 2a,b).

Further standardization is required to use urine HPV viral load as a potential parameter in future studies. Viral load may be expressed as copies of HPV DNA per ml of urine, copies of HPV DNA per µL of DNA extract, copies of HPV DNA per copies of human DNA or copies of HPV DNA per number of cells. The latter expression is used frequently to report viral load in cervical samples. The expression of urinary HPV viral load in urine as copies per ml of urine incorporates variation induced by the volume of collected first-void urine. Figure S1 shows that the collected volumes ranged from 7 to 86 mL without a collection device, which indicates that a urine collection device may be a valid option to standardise the volume of first-void collected urine. Calculation of viral load by dividing the copies HPV DNA by the copies of human DNA detected in urine is somewhat artificial because it also measures trans-renal DNA and captures exfoliated cell debris from the complete genital track in first-void urine. However, we should not exclude this option to account for the amount of human DNA detected in a urine sample to normalise the amount of detected HPV DNA. We asked HPV-infected women to collect eight urine samples at two different time points using two different methods over a four-day period in a separate study. A correlation between the amount of HPV16 DNA and the copies of human DNA in urine was observed in four women in the cohort infected with HPV16 [20] (see also Figure S5). These results confirm that the amount of HPV DNA is linked to the amount of human DNA, which is influenced by the amount of debris from exfoliated cells that contaminates the first-void urine.

The level of cervical sampling reproducibility may also be hindered by the number of cells or human DNA sampled. The type of brush, pressure on the brush, the number of rounds, and the removal of cells from the brush likely influence the type and number of cells that are collected. Differences between physicians are also expected. Two (female) study doctors collected all cervical samples in our study, thus limiting the variability.

Theoretically, knowledge of HPV copies per “infected” cell may be relevant to determining the status of infection (e.g., productive *versus* abortive infection) [25]. However, we cannot differentiate an infected cell from a healthy cell in urine or cervical samples.

The concept of viral load in urine or cervical samples is further hindered by the lack of knowledge of the form of HPV DNA that is detected. HPV DNA can be cell-associated or cell-free and packed or unpacked in viral capsid proteins.

HPV lesions may influence the presence of cellular material, which further adds to the complexity of the viral load concept. Cells in CIN2 or CIN3 lesions express less intercellular adhesion molecules and may be collected more readily using a cytobrush [26]. First-void urine likely also contains more exfoliated cell debris from these lesions, but this hypothesis requires confirmation in future studies.

The value of HPV viral load monitoring requires further investigation. However, we demonstrated that urine samples may have a potential role in this research. A workable definition of viral load likely requires more comprehensive tools to identify the form of HPV DNA (e.g., DNA from viral particles, free HPV DNA, HPV episomes, integrated HPV DNA) before HPV viral load becomes useful for monitoring HPV infections.

### 3.4. Advantages of Urine Sampling over Cervical Sampling

We have previously reported the potential advantages of HPV DNA testing using urine [15,16,27,28]. Urine is a non-invasive sample that may be collected at home and sent by mail to the laboratory for analysis. This methodology provides an important benefit for organizations conducting vaccine and therapeutic trials because of its feasibility. The results of this pilot study using a urine collection device are very promising and provide HPV DNA results that are consistent with previous and subsequent gynaecological samples obtained during a follow-up visit at the clinic.

We recently published the results of urine testing to monitor the impact of HPV vaccination in Bhutan and Rwanda and confirmed the implementability, feasibility and acceptably of urine sampling and testing for monitoring the impact of HPV vaccination [23].

The preference of providing a urine sample over a vaginal self-sample has been reported previously [29,30].

Urine sampling is non-invasive and does not interfere with the natural history of the infection. In contrast, cervical samples obtained by scraping the epithelium create micro-lesions that allow for new infections if infectious virus particles are present or potentially induces an inflammatory reaction.

Urine samples can be collected at home and sent by mail. Therefore, more frequent sampling may be considered.

### 3.5. Limitations of the Study

In this study, urine collection and processing was initiated in July 2012, and we were still optimising the analytical sensitivity of HPV DNA detection using urine. Several improvements that are now recognized were not applied in this study to avoid sampling or processing bias. The volumes of first-void urine collected ranged from 7 to 86 mL (average of 35 mL and median of 31 mL of urine), which may have diluted some of the samples. Figure S1 clearly shows that the volume of the Colli-Pee^TM^-collected first-void urine was smaller and provided a more concentrated sample. The extracted DNA was eluted in 100 µL, which is an unnecessary dilution because 55 µL provided a sufficient volume to perform all required PCRs. A potential additional drawback may be that the women came for a gynaecological examination, which may have led to thorough washing of the genital area and removal of mucus and exfoliated cell debris around the urethral orifice, which was washed away by the first voided urine. We did not take into account the stratified DNA extraction in the function of the cellularity of the samples for the calculation of the cervical HPV DNA copies per µL of DNA extract. We also did not take into account the volume of provided urine for the calculation of the urinary HPV DNA copies per µL of DNA extract. However, the variation in both cases was limited to less than 1 logarithm to base 10 changes. Therefore, the variation exerted only a minor impact on the substantial inter- and intra-participant changes in HPV DNA copies detected. We only optimised an in-house qPCR for HPV16 or HPV18, and we did not test for other HPV genotypes in the urine samples. However, previous studies have confirmed the good correlation between HPV genotypes detected in urine and subsequent cervical samples.

## 4. Materials and Methods

### 4.1. Participants

Eighteen women who were recruited for a phase-one HPV therapeutic vaccine trial were enrolled in the study. The inclusion criteria of the vaccine trial were consistent with our trial. The participants were 22–44 years of age, (average 32.2 years) and had a cervical sample with normal cytology that tested positive for HPV16 and or 18 DNA prior to the start of the vaccine trial. However, the women participating in this study were already in a booster or high-dose arm. Therefore, some women did have ASC-US or were HPV DNA-negative at M0. The vaccine study and the urine collection study were approved by the Ethical Committee of the University Hospital in Antwerp, and all women provided informed consent to participate. Information on the therapeutic trial (vaccine, placebo and or treatment) was blinded to the researchers who examined the urine samples [31].

### 4.2. Collection of Urine Samples

Two different urine collection methods were used. First, the investigator asked the participants to provide a first-void (*i.e.*, initial stream) urine sample of approximately 50 mL in a standard receptacle for urine collection (100 mL) during the different consultations at our centre at specific time points (M0, 2W, 2W + 5D, 4W, 6W, 6M, 12M, 18M, 24M and 3Y (Figure 1)). The volume was recorded. The urine sample was divided into two vials containing a commercial preservation medium (BD ProbeTec™ Urine Preservative Transport Kit, BD Benelux, Erembodegem, Belgium) within 30 min after collection (Figure S6a). The samples were stored at −80 °C until further processing.

Ten participants participated at a small pilot study to evaluate a second urine collection method, *i.e.*, the Colli-Pee^TM^ device (Novosanis, Wijnegem, Belgium), which is designed to collect first-void urine (Figure S6b). The participants self-collected a urine sample at home using this device which is equipped with a collection tube prefilled with 7 mL of a urine-conservation medium. The urine samples were returned by mail to the University of Antwerp. Returned samples were stored at −80 °C until further processing (results shown in Figure S4).

Urine sampling at 3 years (Figure 1, 3Y time point) was performed using an improved Colli-Pee^TM^ device. The participants were invited for a follow-up visit at the University of Antwerp, where they self-collected a 17–20-mL first-void urine sample without urine-conservation medium. Urine samples were placed on ice immediately after collection. Samples were divided into aliquots containing the urine-conservation medium within one hour, mixed and stored at −80 °C until further processing.

### 4.3. DNA Extraction from Urine Samples

DNA extraction was preceded by an ultra-Amicon filtration step to concentrate all DNA. The entire amount in the BD vial (BD ProbeTec™) (3.2 mL of urine or 4 mL of the urine sample plus buffer) that was collected with the Colli-Pee^TM^ device was poured or pipetted onto an Amicon Ultra-4 50 K filter (Merck Millipore, Belgium). The filters were centrifuged at 4000× *g* for 20 min. NucliSENS Lysis Buffer (2 mL) (bioMérieux Benelux, Brussels, Belgium) was added to the concentrate retained on the filter and incubated for 10 min at room temperature. All material was subsequently transferred to the NucliSENS Lysis buffer vial, and DNA extraction was performed on the NuclSENS^®^ easyMag^®^ (bioMérieux) using the off-board lysis protocol. DNA was eluted in 100 µL of elution buffer. Our results are presented as copies/µL DNA extract. A volume of 3.2 mL of urine was first concentrated, and the DNA was subsequently eluted in 100 µL samples. Thus, the DNA copies should be multiplied by a factor of 31.25 or 1.49 logarithm-to-base 10 should be added to the log_10_ copies to determine the copies/mL of urine.

### 4.4. Type-Specific Real-Time Quantitative PCR of DNA Extracted from Urine

The DNA extracts obtained from urine were processed using quantitative amplification and detection of HPV DNA and human DNA with the LightCycler480 Real-Time PCR instrument (Roche Diagnostics Belgium, Vilvoorde, Belgium). The details of the quantitative PCRs for HPV16 (E6), HPV18 (E7) and human DNA (GAPDH) are described elsewhere [19].

### 4.5. Collection of Cervical Samples

The time of collection of cervical samples was M0, 6, 12, 18, and 24, as shown in Figure 1. Gynaecological examinations were performed after the collection of urine samples. Cervical cells were collected into BD-SurePath (BD Diagnostics–Tripath, Burlington, NC, USA) using a Cervex-Brush Combi (Rovers Medical Devices B.V., Oss, The Netherlands). Cytology was assessed in thin-layer cell preparations of cervical cells collected from brush scrapings. Thin-layer cell preparations were stained for cytological analysis and generated using the BD PrepStain slide processor (BD Benelux, Erembodegem, Belgium) using 200 µL of cervical cell-enriched fraction. Thin-layer cytology slides were read without knowledge of the HPV results. Cytological results were classified according to the 2001 version of the Bethesda System [32]: Atypical Squamous Cells of Undetermined Significance (ASC-US), Atypical Squamous Cells of Undetermined Significance—cannot exclude High-Grade Squamous Intraepithelial Lesion (ASC-H), Low-Grade Squamous Intraepithelial Lesion (LSIL), High-Grade Squamous Intraepithelial Lesion (HSIL), Squamous Cell Carcinoma (SCC), and Atypical Glandular Cells (AGC).

### 4.6. DNA Extraction and PCR of Cervical Samples

Processing of the cervical samples has been described in detail elsewhere. Briefly, 8 mL of the 10 mL A SurePath vial (BD Diagnostics–Tripath, Burlington, NC, USA) was converted to 1 mL of liquid-based preparation for cervical sample preparation. A volume of 200 µL of this 1000 µL was used for the liquid-based preparation. Either 400 or all 800 µL of the remaining sample was used for DNA extraction, depending on the cellularity (measured by optical density). The volume of the crude DNA extract ranged from 80 to 250 µL, in which the samples containing more cells were more diluted [33,34].

DNA was isolated from the cell pellet remainder using a JANUS Automated Solution Handling System (Perkin-Elmer, Waltham, MA, USA). TaqMan-based multiplex qPCR (Life Technologies, Carlsbad, CA, USA) was performed to detect HPV genotypes, including HPV16 (E6) and HPV18 (E7), and β-globin was used as a control [34,35].

We transformed the laboratory-reported HPV copies per cervical cell to the number of HPV DNA copies per µL of DNA extract of the cervical sample to compare the urine HPV results with the cervical HPV results. Therefore, we multiplied the copies per cell by the total number of cells present in the sample. This calculation does not account for the stratified dilution according to the cellularity during the DNA extraction of the liquid-based cytology sample, but it does provide an order of magnitude.

### 4.7. Statistics and Software

All statistical analyses (κ agreement, Spearman rho) and graphics were performed using SPSS statistics version 22 (IBM Corporation, Armonk, NY, USA).

## 5. Conclusions

We demonstrated that standardized urine sampling may be a valid option for the follow-up of persistent infections or persistent clearance in a therapeutic HPV vaccine trial. Non-invasive and at-home sampling may substantially simplify the operational management of an HPV vaccination trial and may increase the sampling frequency if required.

The natural history of an HPV infection may impact the amount of viral HPV DNA detected, but the potential clinical value of urine viral load monitoring should be further investigated.

## Figures and Tables

**Table 1:** Overview of the quantitative PCR results for each of the analyzed parameters, *i.e.*, HPV16, HPV18 and human DNA (hDNA) in urine and cervical samples (Cervix).

Case Summaries						
Sample Type and DNA Tested	Urine HPV16	Cervix HPV16	Urine HPV18	Cervix HPV18	Urine hDNA	Cervix hDNA
*N* (number of samples) *	124	60	66	32	172	85
Mean (copies/µL) **	7474	43,566,170	43	27,670	10,983	3081
Median (copies/µL) **	140	74,826	0	364	5300	2018
Minimum (copies/µL) **	0	0	0	0	15	44
Maximum (copies/µL) **	267,000	752,829,409	989	394,818	59,800	19,559

* All samples were tested for hDNA. The sum of the HPV16 plus HPV18 tests was higher than the total number of samples collected because of the co-infections observed in numerous participants. The urine samples from the pilot study are also included; ** The amount of detected DNA is expressed as copies/µL DNA extract.

**Table 2:** Urine HPV16 *versus* cervical HPV16 positivity. κ: 0.683, *p* < 0.001.

		Cervical HPV16		Total
		Negative	Positive	
Urine HPV16	Negative	11	5	16
	Positive	2	42	44
Total		13	47	60

**Table 3:** Urine HPV18 *versus* cervical HPV18 positivity. κ: 0.510, *p* = 0.002.

		Cervical HPV18		Total
		Negative	Positive	
Urine HPV18	Negative	10	7	17
	Positive	1	14	15
Total		11	21	32

## Supplementary Materials

Supplementary materials can be found at http://www.mdpi.com/1422-0067/17/5/750/s1.

## Abbreviations

AGC	Atypical Glandular Cells
ASC-H	Atypical Squamous Cells cannot exclude High-grade Squamous Intraepithelial Lesions
ASC-US	Atypical Squamous Cells of Undetermined Significance
CIN	Cervical intraepithelial neoplasia
GAPDH	Glyceraldehyde-3-phosphate dehydrogenase
hDNA	Human DNA
HPV	Human Papillomavirus
hrHPV	High-Risk Human Papillomavirus
HSIL	High-Grade Squamous Intraepithelial Lesions
IARC	International Agency for Research on Cancer
LSIL	Low-Grade Squamous Intraepithelial Lesions
NILM	Negative for Intraepithelial Lesion or Malignancy
PAP	Papanicolaou
PCR	Polymerase Chain Reaction
qPCR	Quantitative PCR
WHO	World Health Organisation
