# Supplementary material for: Long-Term Follow-up of HPV Infection Using Urine and Cervical Quantitative HPV DNA Testing
Source: Int J Mol Sci. 2016 May 17;17(5):750. doi: 10.3390/ijms17050750 (PMC4881571; doi:10.3390/ijms17050750)
Supplement: Supplementary file 1 [file ijms-17-00750-s001.pdf]

# Supplementary Materials: Long-Term Follow-up of HPV Infection Using Urine and Cervical Quantitative HPV DNA Testing

**Alex Vorsters, Severien Van Keer, Samantha Biesmans, Annick Hens, Ilse De Coster, Herman Goossens, Margareta Ieven and Pierre Van Damme**

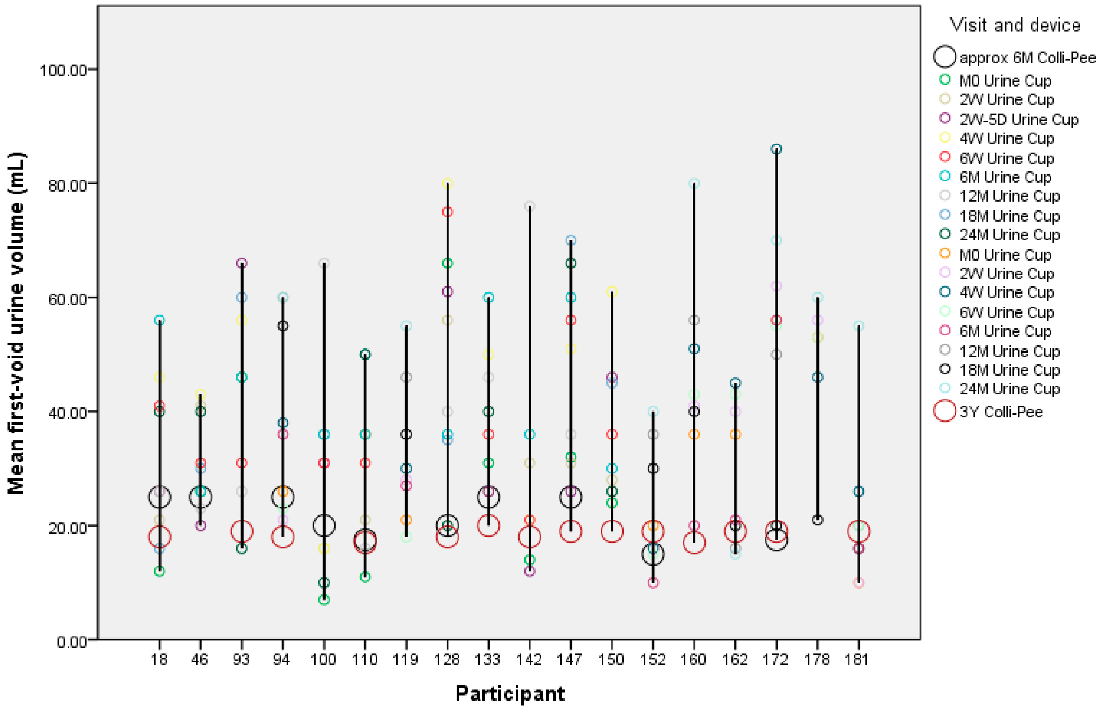


**Figure S1.** Variation of collected urine volumes per participant, visit and collection device. Visits are defined by time of sampling: M0 (month 0, start of the trial), W: week, D: days, Y: years.

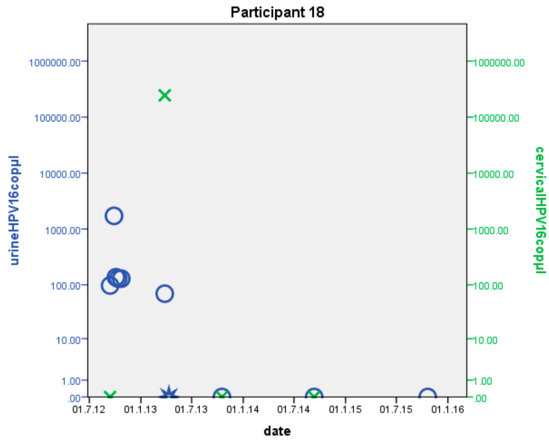


(**a**)

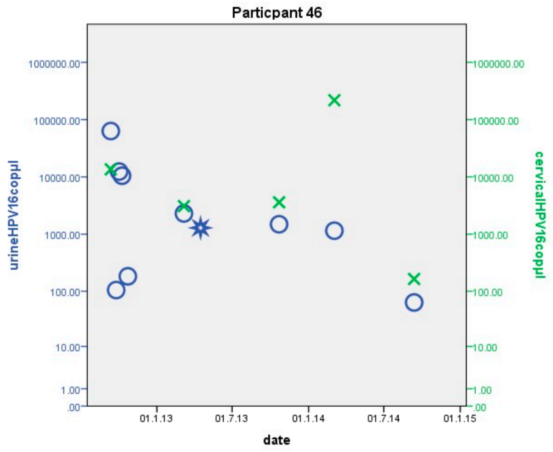


(**b**)

**Figure S2.** *Cont.*

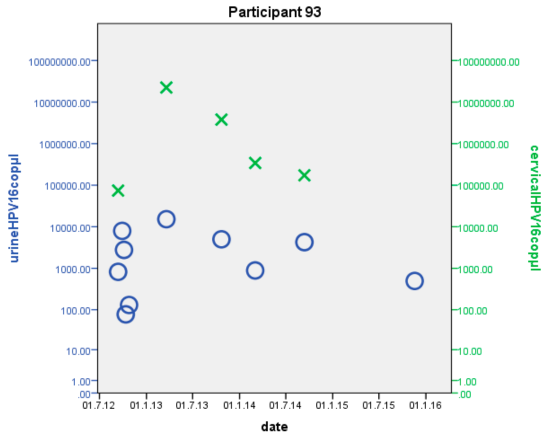


(**c**)

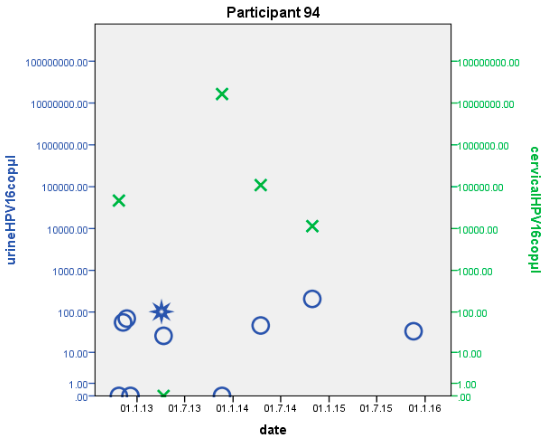


(**d**)

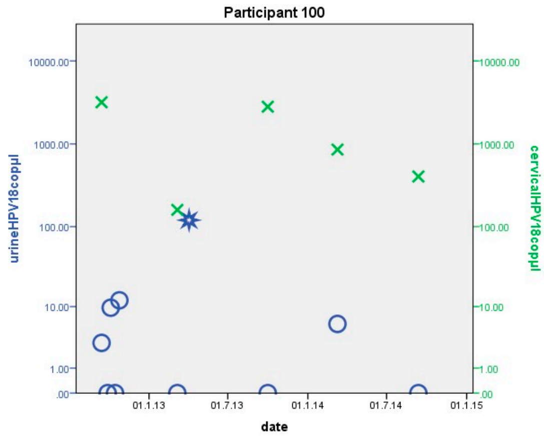


(**e**)

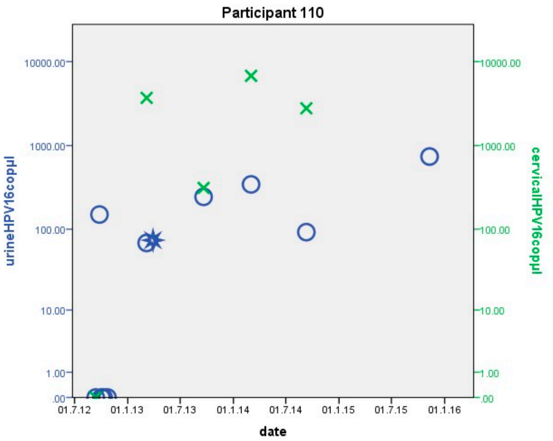


(**f**)

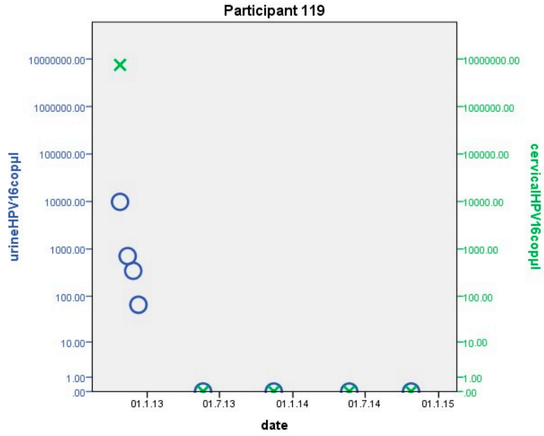


(**g**)

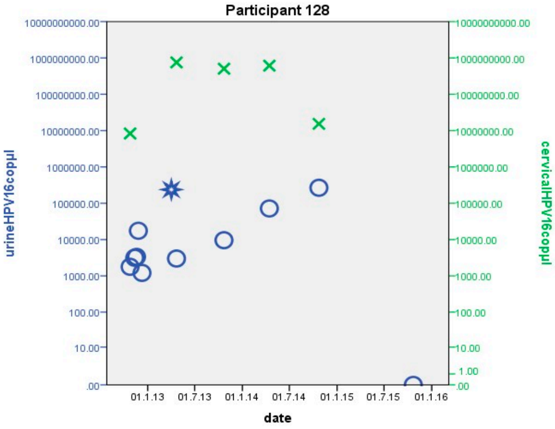


(**h**)

**Figure S2.** *Cont.*

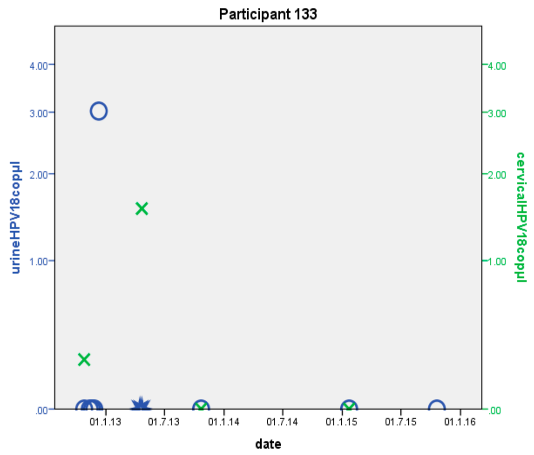


(**i**)

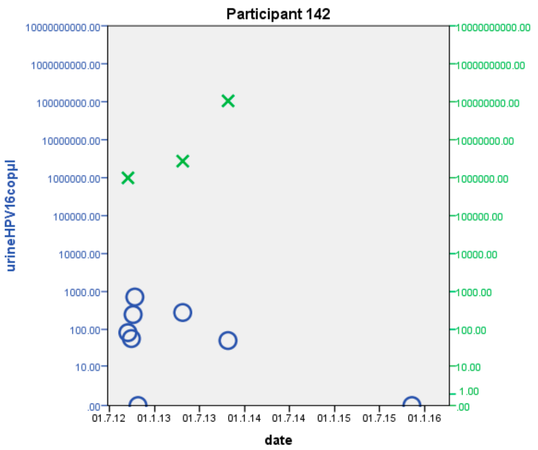


(**j**)

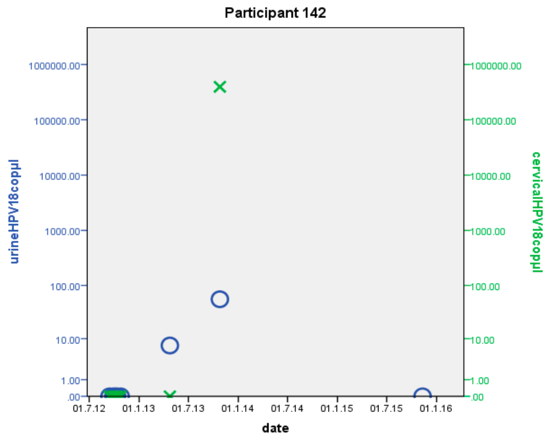


(**k**)

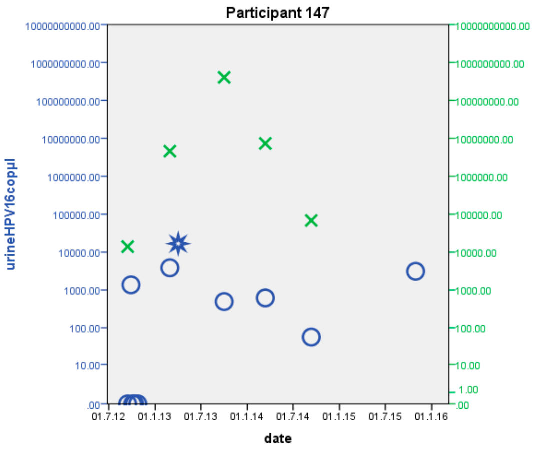


(**l**)

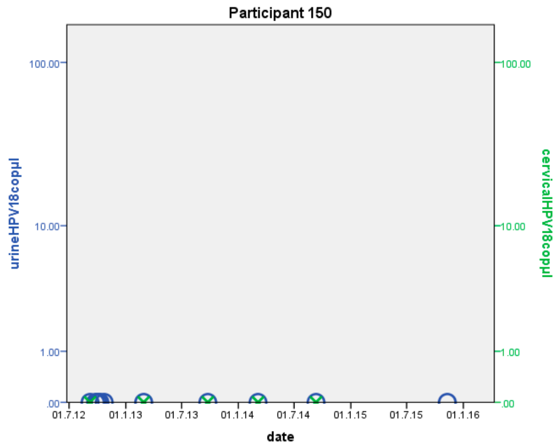


(**m**)

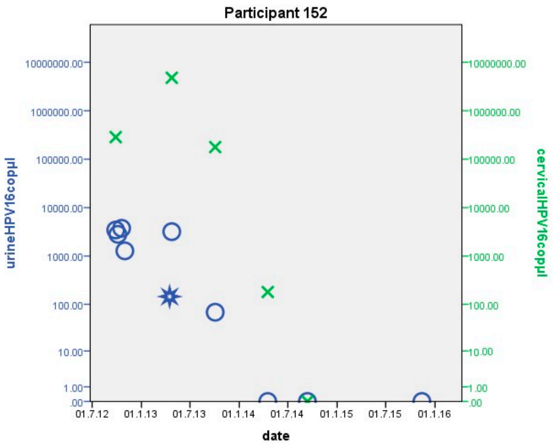


(**n**)

**Figure S2.** *Cont.*

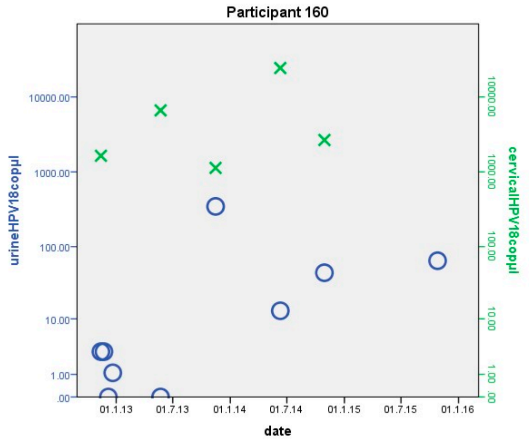


(**o**)

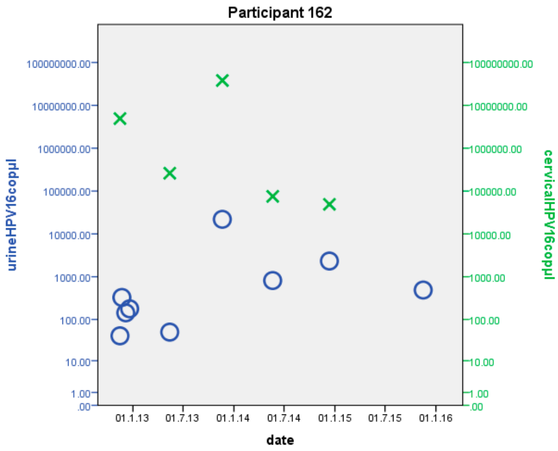


(**p**)

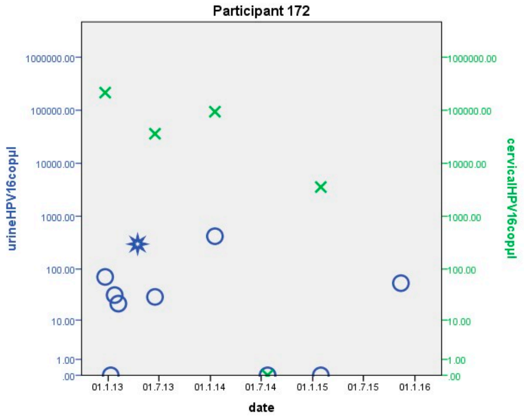


(**q**)

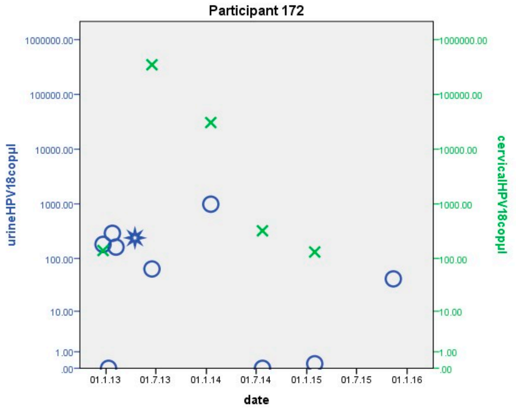


(**r**)

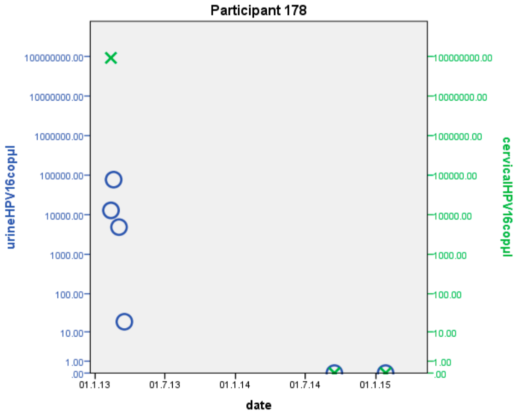


(**s**)

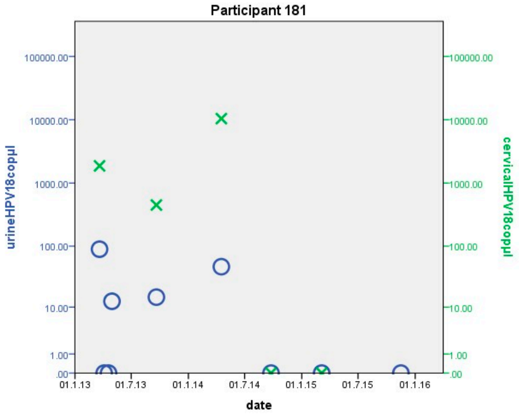


(**t**)

**Figure S2.** (**a**–**t**) Individual plots of HPV copies found in urine and cervical samples over 2–3 years. Time points on the *x*-axis are dates of the different visits (M0 to M24 or 3 Y). ×, O, ✷ labels indicate different sample types, ×: cervix; O: urine; ✷: home collected urine (Colli-Pee™). Left *y*-asix copies HPV/μL in urine sample, Right *y* -axis copies HPV/μL in cervical sample.

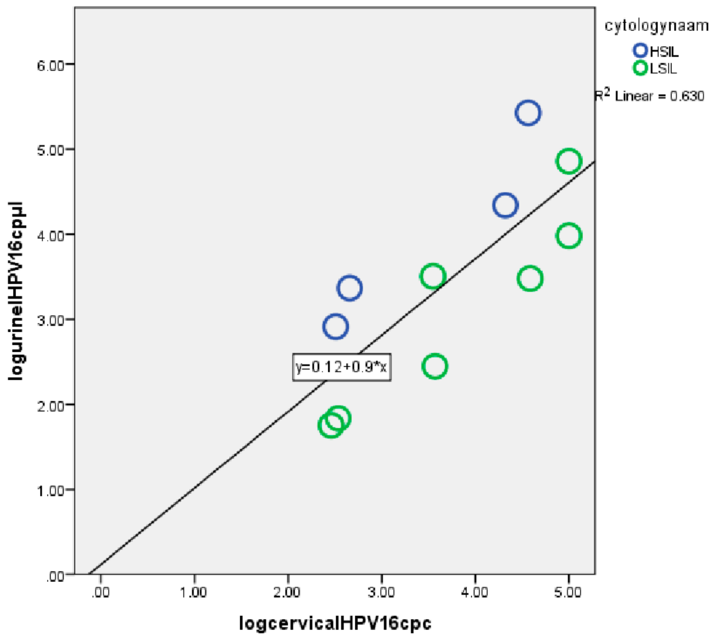


**Figure S3.** Correlation between HPV16 copies found in urine versus HPV16 copies found in cervical material.

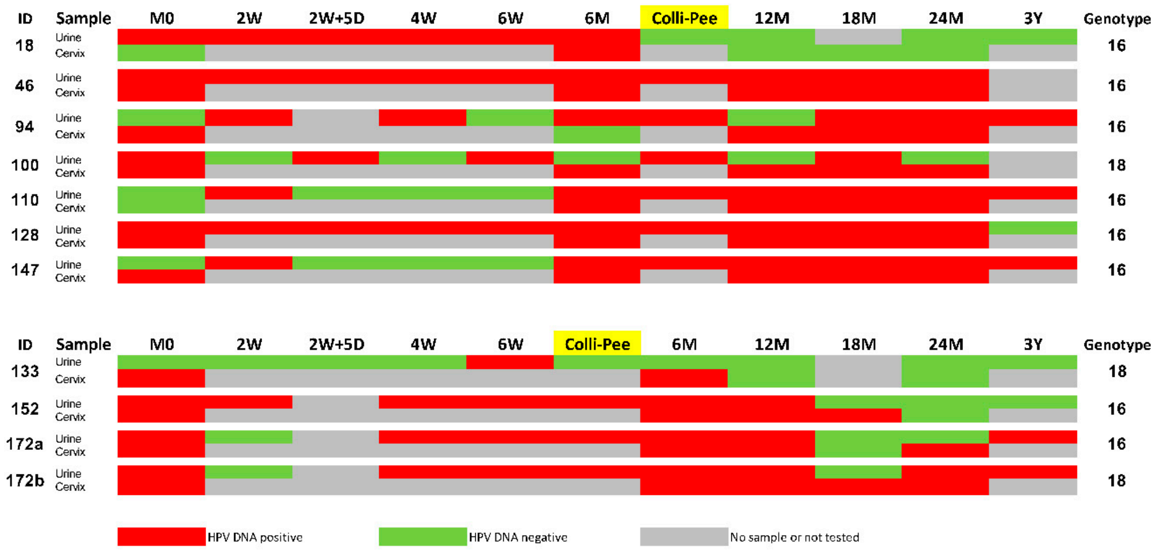


**Figure S4.** Results from home-collected urine sample compared to the cervical and clinic-collected urine samples.

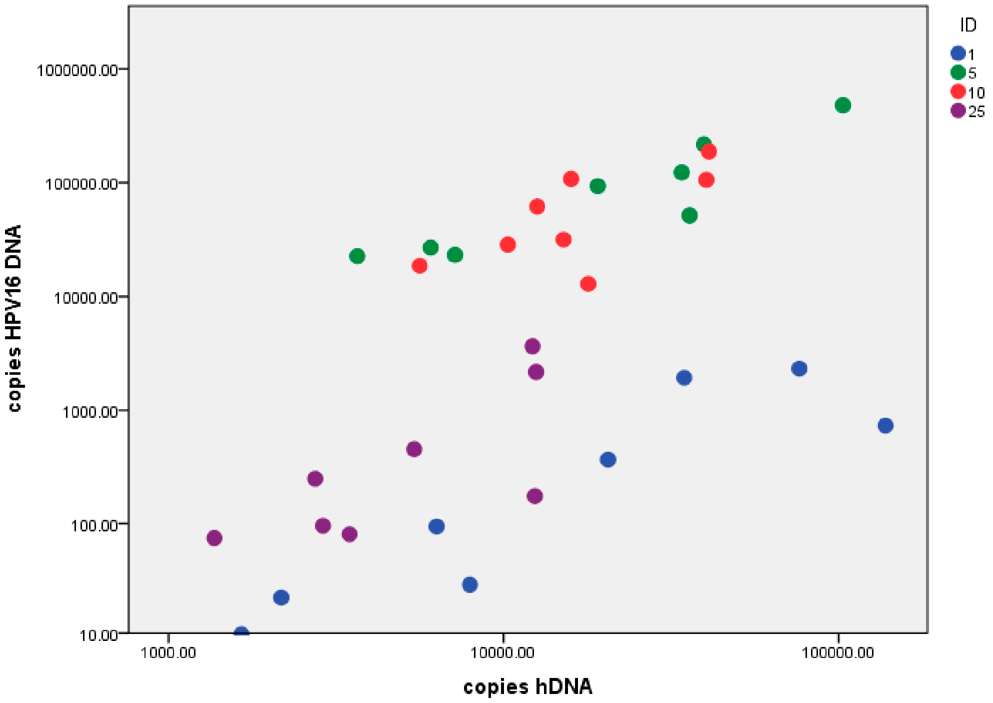


**Figure S5.** Dot plot of HPV DNA copies versus human DNA copies from 8 urine samples provided by 4 women on 4 consecutive days [16].

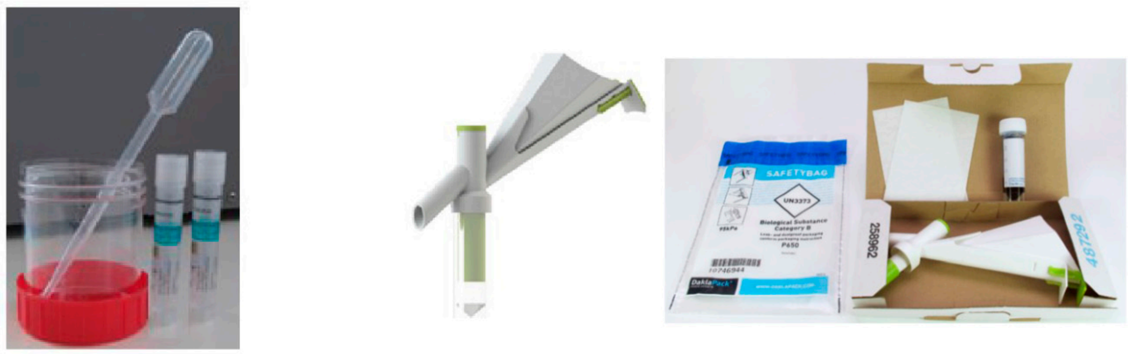

a) b)

**Figure S6.** (**a**,**b**) Picture of the different urine collection methods. Standard urine recipient and Colli-Pee™.
